# A Query

**Published:** 1885-04

**Authors:** O. W. Smith

**Affiliations:** Union Springs, N. Y.


### A QUERY.

ED. HOMŒOPATHIC PHYSICIAN:—What does the author of "Who is our Guide, and Where Shall We Find Him?" mean by his statement (p. 75)? Lippe's Repertory has under "*fetid perspiration* of feet, Amm. carb., Amm. mur., Bar.-c., Cycl., Graph., Kal.-c., Kobalt, Nit. ac., Phos., Plumb., Sep., Sil., Thuj., Zinc., and Allen's Repertory has under *Sweat of an offensive odor*, Arund., Sep., Sil., Tel., Wies., Zinc. Lippe gives *five* remedies more than Guernsey, and Allen *one* more. "Be sure you're right," then *write*. O. W. SMITH, M. D.

UNION SPRINGS, N. Y.

